# Biotemplating rod-like viruses for the synthesis of copper nanorods and nanowires

**DOI:** 10.1186/1477-3155-10-18

**Published:** 2012-05-01

**Authors:** Jing C Zhou, Carissa M Soto, Mu-San Chen, Michael A Bruckman, Martin H Moore, Edward Barry, Banahalli R Ratna, Pehr E Pehrsson, Bradley R Spies, Tammie S Confer

**Affiliations:** 1 Naval Research Laboratory, Center for Bio/Molecular Science and Engineering, 4555 Overlook Ave. S.W., Washington DC, 20375, USA; 2 Martin Fisher School of Physics, Brandeis University, 415 South St., Waltham, MA, 02454, USA; 3 Naval Research Laboratory Code 6100, 4555 Overlook Ave. S.W., Washington DC, 20375, USA; 4 Naval Research Laboratory Code 5711, 4555 Overlook Ave. S.W., Washington DC, 20375, USA; 5 NRL/NRC postdoc resident at Naval Research Laboratory, Washington DC, USA; 6 Present address: IBM Almaden Research Center, 650 Harry Rd, San Jose, CA, 95120-6099, USA

**Keywords:** Tobacco mosaic virus, M13 phage, *fd* phage, Electroless deposition, Polyaniline coating, Dispersion

## Abstract

**Background:**

In the past decade spherical and rod-like viruses have been used for the design and synthesis of new kind of nanomaterials with unique chemical positioning, shape, and dimensions in the nanosize regime. Wild type and genetic engineered viruses have served as excellent templates and scaffolds for the synthesis of hybrid materials with unique properties imparted by the incorporation of biological and organic moieties and inorganic nanoparticles. Although great advances have been accomplished, still there is a broad interest in developing reaction conditions suitable for biological templates while not limiting the material property of the product.

**Results:**

We demonstrate the controlled synthesis of copper nanorods and nanowires by electroless deposition of Cu on three types of Pd-activated rod-like viruses. Our aqueous solution-based method is scalable and versatile for biotemplating, resulting in Cu-nanorods 24–46 nm in diameter as measured by transmission electron microscopy. Cu^2+^ was chemically reduced onto Pd activated tobacco mosaic virus, *fd* and M13 bacteriophages to produce a complete and uniform Cu coverage. The Cu coating was a combination of Cu^0^ and Cu_2_O as determined by X- ray photoelectron spectroscopy analysis. A capping agent, synthesized *in house,* was used to disperse Cu-nanorods in aqueous and organic solvents. Likewise, reactions were developed to produce Cu-nanowires by metallization of polyaniline-coated tobacco mosaic virus.

**Conclusions:**

Synthesis conditions described in the current work are scalable and amenable for biological templates. The synthesized structures preserve the dimensions and shape of the rod-like viruses utilized during the study. The current work opens the possibility of generating a variety of nanorods and nanowires of different lengths ranging from 300 nm to micron sizes. Such biological-based materials may find ample use in nanoelectronics, sensing, and cancer therapy.

## Background

A great interest in gold nanorods is being motivated by their potential applications in various technologies including optical filtering, subwavelength imaging, data storage, and sensor devices [1,2]. One efficient approach in preparing large quantities of gold nanorods is via seed-mediated synthesis in the presence of a surfactant [3]. Nonetheless, polydispersity and byproducts in the form of nanospheres and nanoplates may be limiting for certain optical applications. Recent progress in purification [4] and synthesis of gold-nanorods [5] shows great promise. Moreover, interest in nanorods of various metals had arisen as well. For example, copper oxide nanorods had been produced by Liu *et al.*[6] and Cheng [7], who were motivated by potential applications of copper oxide nanorods in solar and electrochemical cells. While their methods have potential for the production of copper oxide nanorods on surfaces, scalable solution-based methods that produce monodisperse and well-dispersed copper (Cu) nanorods is still a topic of significant importance in fundamental studies and commercial applications.

Our strategy for the synthesis of Cu-nanorods is biotemplating. Biotemplating is an attractive method to synthesize nanosize inorganic materials because it takes advantage of the well-defined size and shape of the biological structures and the surface functional groups that can interact with metal atoms leading to nucleation and growth of nanoparticles. It can potentially produce a wide variety of materials for applications in electronics, sensing, optics, and cancer therapy [8-10].

Biomolecules such as DNA, amino acids, peptides, protein cages, and viruses have been used as templates and scaffolds for the synthesis of inorganic nanomaterials including metals and semiconductors [11]. DNA [12] and fiber-like protein structures like microtubules [13] have been used as biotemplates for the synthesis of Cu-nanowires. Still the utilization of DNA and microtubules for biotemplating face some challenges. For example, DNA requires specialized techniques for the production of straight nanowires and the aspect ratio of Cu-nanowires from microtubules is difficult to control due to the polydisperse nature of the microtubules.

Rod-like viruses provide the following advantages for the synthesis of nanorods: (1) well-define shape and dimensions in the nanoscale, (2) stability at broad pH ranges, (3) easy to purify in large scale, (4) mechanically robust, which allows the utilization of ultracentrifugation and sonication techniques during sample processing, and (3) virus particles are intrinsically monodisperse. The rod-like plant virus tobacco mosaic virus (TMV) and bacteriophages like *fd* and M13 are ideal templates for producing high aspect ratio materials such as nanorods. These viruses also share common favourable characteristics for biotemplates including stability over a wide pH range, and a net negative charge at neutral pH. TMV is a 300 nm long cylindrical rod with an outer diameter of 18 nm and a 4 nm central cavity. Approximately, 2130 identical coat protein (CP) subunits form a right-handed helix around the viral single stranded RNA [14,15]. Filamentous bacteriophage *fd* and wild type M13 are structurally identical. They are 880 nm in length and 6.6 nm in diameter [16]. Each phage consists of approximately 2700 CP (pVIII) subunits wrapped around a circular loop of single stranded DNA. *fd* and M13 differ by one amino acid per CP, which results in a net 30% more negative charge in *fd*[17,18]. The M13 phage is a widely-used cloning system as a phage display for expression of small peptides [19] used to identify amino acid sequences that are specific towards metals, metal oxides [20], and semiconductor surfaces [21].

High aspect ratio viral protein structures have been explored to fabricate metallic nanorods. Even though a variety of metals have been deposited on TMV [22-35] and M13 [36,37], continuous coating have been reported only for Pd [27], Pt [31], Co [32], and Ni [32,33] on TMV and Ag [36] and Au/Ag alloy [37] on M13. Meanwhile, there are not reports to date on the metallization of the *fd* bacteriophage.

Among the various metals, Cu offers the advantages of high electric conductivity and low cost. If high quality biotemplated Cu-nanorods and Cu-nanowires can be fabricated in large quantities, they may be of utility as interconnects in future nanoscale electronics [38]. Previous strategies for copper incorporation into TMV include photochemical reduction of Cu^2+^ TMV [39], direct chemical reduction [34] of CuCl_2,_ and copper reduction inside the TMV channel [35]. Major issues reported in the literature include sparse and uneven Cu coverage [39], product aggregation [34], poor yield and difficulties in controlling the length of the resulting Cu-nanorods.

In the current work, we report the synthesis of straight, continuous and dispersed Cu-nanorods and Cu-nanowires by electroless deposition of Cu on Pd-activated virus outer surfaces. Our solution-based method is performed in aqueous solution and at room temperature, making it amenable for large scale production. The Cu-TMV nanorods were characterized using transmission electron microscopy (TEM), scanning electron microscopy (SEM), X-ray photoelectron spectroscopy (XPS), and a nanoparticle size determination system. Furthermore, we produced Cu-*fd* and Cu-M13 nanorods and PANI-Cu-TMV nanowires to demonstrate the versatility of this metallization procedure for other biotemplates. The current work opens the possibility of generating a variety of nanorods and nanowires of different lengths ranging from 300 nm to micron sizes.

## Results and discussion

### Electroless deposition of Pd on wild type (WT) TMV

The synthesis of TMV-templated Cu-nanorods was achieved by a two-step electroless deposition on wild type (WT) TMV. In the first step, the virus template surface was activated by seeding small Pd nanoparticles on the surface. In the second step, the Pd nanoparticles served as catalytic sites for the chemical reduction of Cu^2+^, leading to nanocrystal growth and the formation of continuous Cu coating on the template.

**Figure 1:**
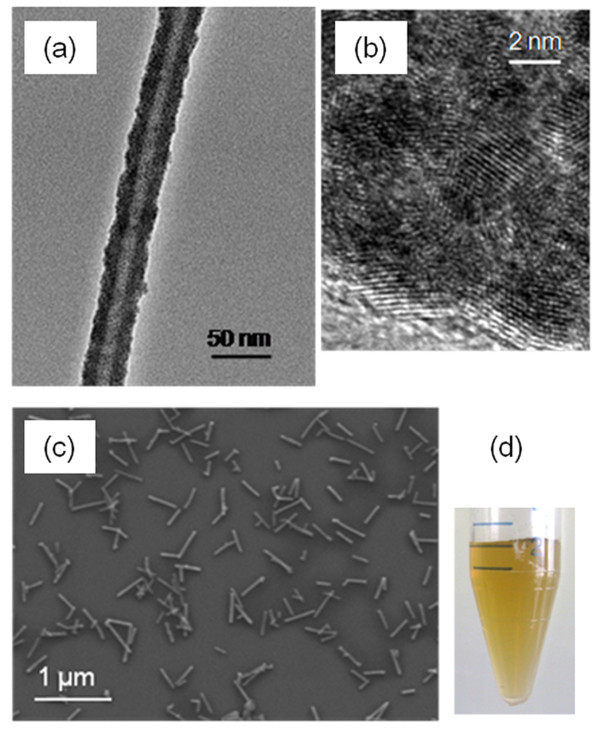
**Images of Pd-TMV produced with 0.4 mM Na**_**2**_**Pd**_**2**_**Cl**_**4**_**.** (**a**) TEM image of a Pd-TMV. (**b**) Higher resolution image from (**a**) white region correspond to the grid and the dark area to the Pd-TMV. (**c**) SEM image of Pd-TMV dispersed on Si wafer. (**d**) Picture of a centrifuge tube containing uniformly colored Pd-TMV suspension.

During the Pd activation step, hydrolysis of PdCl_4_^2-^ produced a 2–3 nm colloidal chloro- and hydroxybridged Pd^2+^ species [40], which adsorbed onto the surface of the viruses and formed a densely and continuously packed coating (Figures 1a-b). We found that using TMV within a week after purification and performing buffer exchange to water just prior to Pd reactions is critical to obtain continuous and smooth coating. The resulting Pd-TMV nanorods were straight and uniform in thickness with an average diameter of 35 ± 4 nm. We achieved high quality Pd coverage on WT-TMV after one round of Pd deposition. Aggregation of the Pd coated TMV was prevented by sonicating the solution in presence of ethylenediaminetetraacetic acid (EDTA). SEM image of well dispersed Pd-TMV nanorods on a Si wafer is shown in Figure 1c. Most of the Pd-TMV nanorods were structurally intact with length of 305 ± 119 nm. Some Pd-TMV nanorods were broken as a result of sonication, and some are longer than 300 nm due to the head-to-tail self-assembly tendency of the TMV [41,42]. Pd-TMV treated with EDTA and collected by centrifugation were resuspended in water without visible aggregation (Figure 1d) while Pd-TMV not treated with EDTA (data not shown) resulted in nanorods that did not resuspend in water after centrifugation.

### Electroless deposition of Cu on WT-TMV

**Figure 2:**
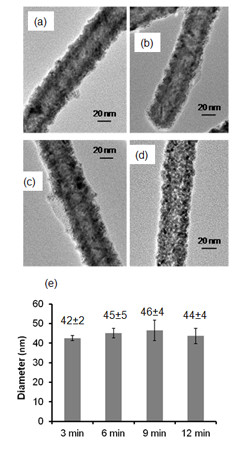
**Representative TEM images of Cu-TMV during time course experiment.** Reaction performed in Cu plating bath containing 1 mM CuSO_4_, 0.03 mM EDTA, and 1 mM DMAB. The Cu-TMV samples were taken from the plating bath (**a**) 3 min, (**b**) 6 min, (**c**) 9 min, and (**d**) 12 min after the reaction started. Numbers on top of the bars correspond to average diameters in nm along with corresponding standard deviations measured from TEM images (n values for standard deviations from left to right: 11, 79, 85, 58, 106).

Electroless Cu plating was performed by mixing 1 μg/ml Pd-TMV into a Cu plating bath containing 1 mM Copper (II) sulfate (CuSO_4_), 0.03 mM EDTA, and 1 mM dimethylamine borane (DMAB). After mixing Pd-TMV solution with Cu plating bath, the mixture turned grey almost instantly. For optimization purposes in early experiments, reactions were run for 12 minutes (Figure 2). Samples were taken from the reaction mix every 3 minutes, and water was added in equal amounts to stop the reaction. The Cu-TMV rods were collected by centrifugation and examined by TEM. TEM images showed straight and continuously coated Cu-TMV nanorods (Figure 2). The average diameter of Cu-TMV nanorods were found to be 42 ± 2 nm, after 3 min of Cu metallization. During the time course experiment (Figure 2) after 6 min reaction time, the change in diameter is not significant. Therefore, reaction time was set to 6 min. Subsequent repeats of Cu-TMV preparations keeping the reaction time to 6 min resulted in an average diameter of 46 ± 5 nm. No byproducts e. g. non-templated nanoparticles were identified in purified samples. Assuming the Pd and Cu layers are cylindrical sheets wrapped around the outside of the TMV, the thickness of the Cu layer calculated as (D_Pd-TMV_ – D_Cu-TMV_)/2 was on average 6 ± 3 nm.

### XPS characterization of Pd-TMV and Cu-TMV

**Figure 3:**
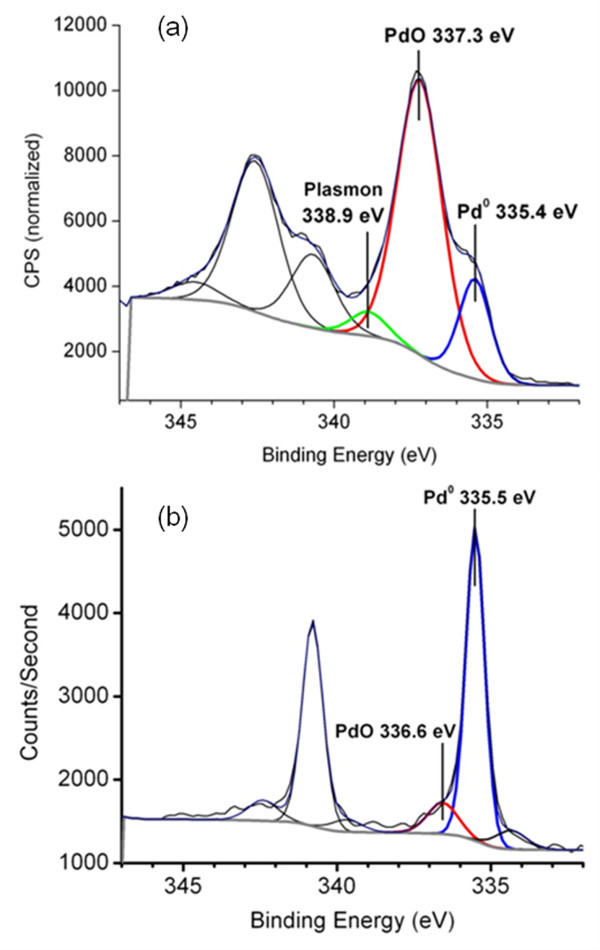
**XPS spectra.** (**a**) Pd-TMV and (**b**) Cu-TMV showing the Pd3d_3/2_ and Pd3d_5/2_. The spectra are fitted with three peaks corresponding to Pd^0^, PdO, and Pd^2+^ shake satellites.

In order to determine the composition and oxidation states of the metals on Pd-TMV and Cu-TMV, we characterized the metal-nanorods via XPS. Peak-fitting of the Pd 3d spectrum for Pd-TMV, shown in Figure 3a, reveals three peaks, a major peak at 337.3 eV, a smaller peak at 335.4 eV, and a minor peak at 338.9 eV, corresponding to the PdO, Pd metal, and Pd^2+^ shake satellites/plasmons, respectively [43-45]. The peaks are similar to those obtained by Lim *et al.*, for Pd-TMV [27]. The Pd spectrum from Cu-TMV (Figure 3b) differs in that it is dominated by the Pd metal peak at 335.5 eV, with a much smaller PdO peak at 336.6 eV. The ratio of PdO to Pd from Pd-TMV is 3.9, i.e. it is mostly PdO, whereas Cu-TMV has a PdO/Pd ratio of 0.16, i.e. it is mostly metallic Pd. The reduction of Pd^2+^ to Pd^0^ was expected as Pd^0^ is the catalyst for the Cu reduction [35]. Pd^2+^ was most likely reduced by the reducing agent DMAB in the Cu plating bath before Cu reduction happened [40]. Similar XPS results were also obtained by others from surface-bound Pd nanoparticles before and after Cu deposition using formaldehyde as reducing agent [46].

**Figure 4:**
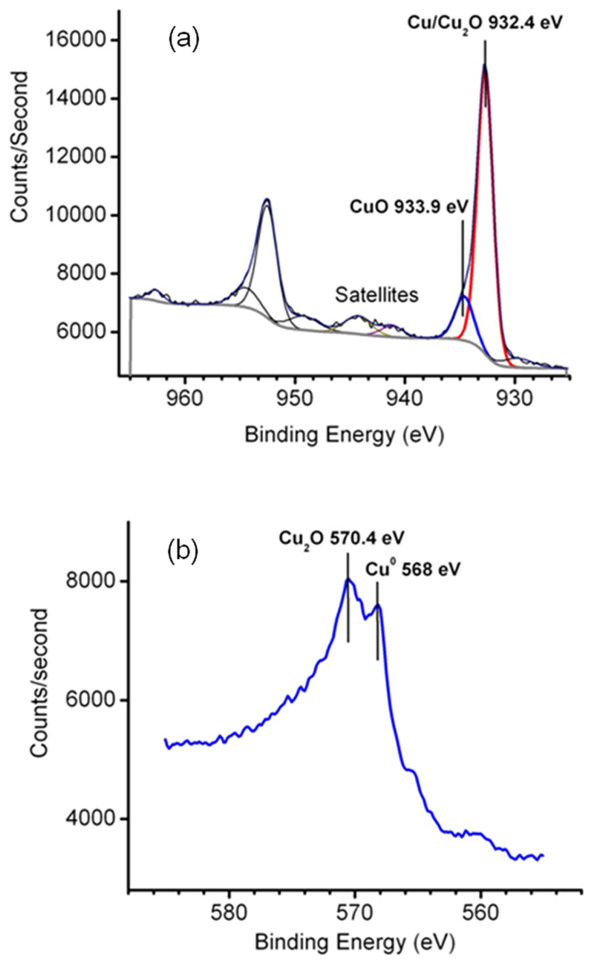
**XPS spectra of Cu-TMV.** (**a**) Cu2p peaks. (**b**) CuL_3_M_45_M_45_ Auger peaks.

The Cu2p_1/2_ spectra from Cu-TMV show a narrow peak at 932.4 eV (Figure 4a), which corresponds to the binding energy of Cu or Cu_2_O [47-49]. Peak fitting reveals that there is only a small contribution from CuO, whose Cu2p_1/2_ peak occurs at 933.2-933.8 eV, with characteristic CuO shake satellites at 940–943.5 eV [48]. The Cu2p_1/2_ binding energies of Cu and Cu_2_O differ by only 0.1 eV, so the Cu2p_1/2_ peak at 932.4 eV cannot be used to resolve the two species, and the L_3_M_45_M_35_ Auger peaks must be used. The L_3_M_45_M_35_ Auger spectra in Figure 4b are dominated by a peak at 570.4 eV, close to the reported value of 569.9 eV and 570.0 eV for Cu_2_O [47,49]. A smaller peak at 568.0 eV represents metallic Cu [47,49]. These results indicate that the Cu coating on the TMV consists primarily of Cu and Cu_2_O.

### Dispersion of Cu-TMV

**Figure 5:**
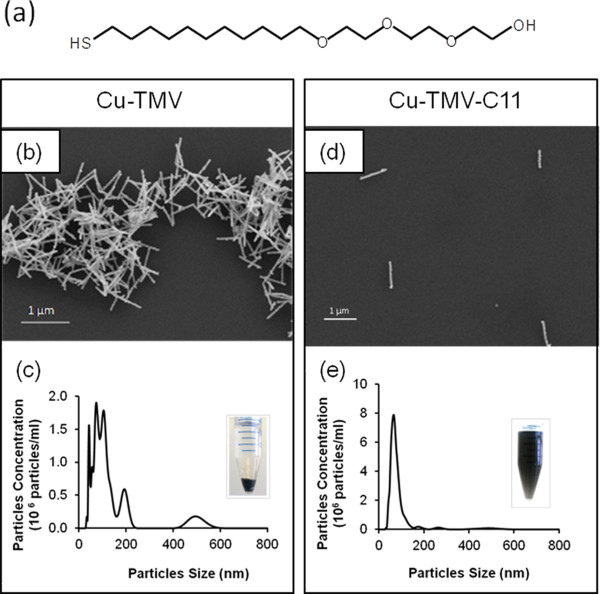
**Dispersion of Cu-TMV, 16 hr after synthesis.** (**a**) Chemical structure of C11-PEG-SH. (**b**) SEM image and (**c**) size distribution of Cu-TMV determined by Nanosight nanoparticle size determination system. (**d**) SEM image and (**e**) size distribution of Cu-TMV-C11 from Nanosight nanoparticle size determination system. Inserts: pictures of the samples 5 min after agitating the tubes. Data demonstrates the successful dispersion of the Cu-TMV after treatment with C11-PEG-SH.

Based on visual examination, Cu-TMV was well-suspended during the Cu plating reaction. Nonetheless after centrifugation, which facilitates Cu-TMV recovery in a scalable fashion, Cu-TMVs were slightly compressed into visible aggregates. In order to re-disperse the Cu-TMV nanorods, we used a capping molecule synthesized in our laboratory [50]. The molecule C11-PEG-thiol consists of an end thiol group linked to a C11 alkyl chain and then an oligo-ethylene glycol (PEG) (Figure 5a). The thiol group interacts strongly with Cu nanocrystals while the C11-PEG chains interact with water. After incubating Cu-TMV (Figures 5b-c) with C11-PEG-thiol at room temperature (RT) overnight, the Cu-TMV nanorods formed a fine black layer at the top. When the solution was gently mixed, the powder resuspended quickly forming a uniformly-colored dispersion (Figures 5d-e). SEM images of the Cu-TMV dried on Si wafers showed individual and small clusters (< 2 μm) of Cu-nanorods in the samples with C11-PEG-thiol (Cu-TMV-C11) (Figure 5d). The control sample without the additive remained as large aggregates that precipitated out from the suspension within a few minutes (Figures 5b-c).

The effect of C11-PEG-thiol was further analyzed using a NanoSight nanoparticle size determination system, which uses laser scattering to track the Brownian motion of individual nanoparticles and calculates the hydrodynamic diameter of the nanoparticles. The software Nanosight NTA2.1 was designed to determine the size of spherical particles; therefore, the length of rod-shaped TMV obtained from the analysis cannot be reported quantitatively and was used only for qualitative comparison. Particle size analysis showed multiple peaks and broad distribution in unmodified Cu-TMV (Figure 5c), but a single sharp peak with an average value of 75 ± 3 nm (n = 3) for Cu-TMV-C11 (Figure 5e). The size distribution of Cu-TMV-C11 was comparable to that of WT-TMV, (Additional file 1: Table S1; Figure S1). C11-PEG-thiol treated Cu-TMV remained stable and readily dispersed at RT for months. Due to the amphiphilic nature of the C11-PEG chain, Cu-TMV-C11 was easily suspended in water and organic solvents such as ethanol and DMSO.

### Metallization of *fd* and M13 bacteriophages

**Figure 6:**
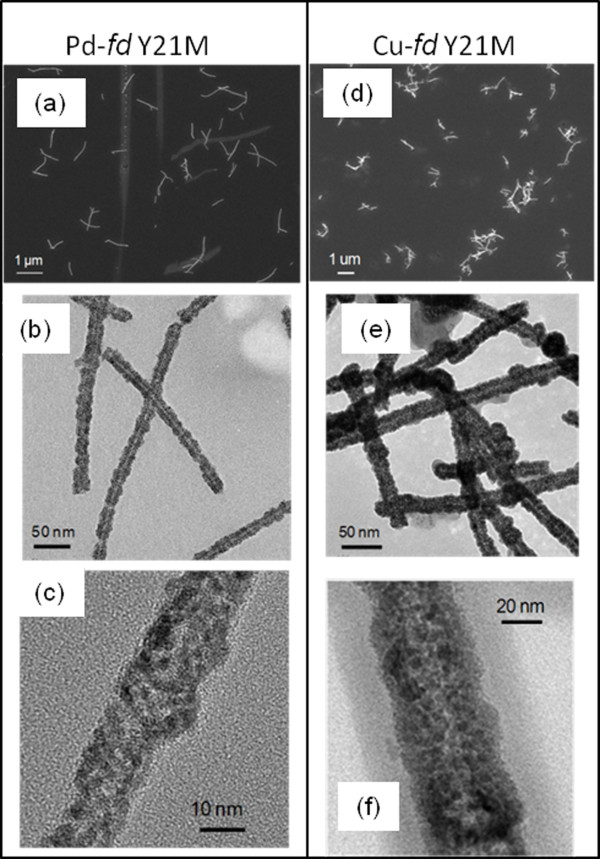
**Images of metalized*fd*Y21M.** (**a**) SEM of Pd-*fd* Y21M, (**b**) and (**c**) TEM of Pd-*fd* Y21M. (**d**) SEM of Cu- *fd* Y21M, (**e**) and (**f**) TEM of Cu- *fd* Y21M.

**Figure 7:**
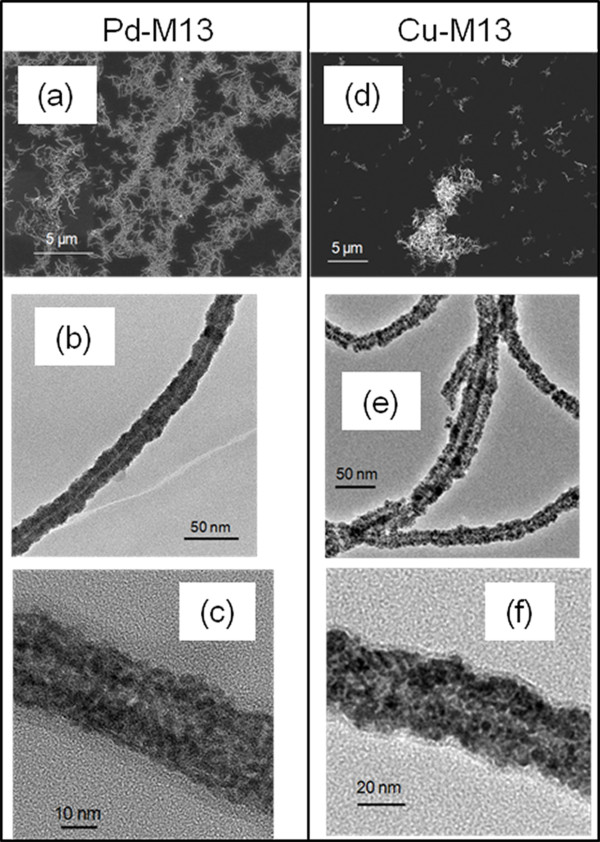
**Images of metalized M13.** (**a**) SEM of Pd-M13, (**b**) and (**c**) TEM images of Pd-M13. (**d**) SEM images of Cu-M13, (**e**) and (**f**) TEM images of Cu-M13.

*fd* mutant Y21M was used for metallization for the first time. The procedure described in previous sections for the fabrication of high quality and dispersed Cu-TMV nanorods was further applied to metalize *fd* and M13 phages (Figures 6 and 7). The bacteriophages are thinner, longer and more flexible than TMV nanorods, with *fd* Y21M mutant being stiffer than M13 [51,52]. Similar to Pd-TMV, Pd-*fd* Y21M and Pd-M13 were densely and continuously coated with 2–3 nm Pd nanoparticles (Figures 6c and 7c). The average diameters of Pd-*fd* Y21M and Pd-M13 were 20 ± 3 nm and 23 ± 4 nm respectively. TEM analysis indicates that Pd-M13 nanorods were slightly more entangled than the Pd-*fd* Y21M, (Figures 6a and 7a) reflecting the more flexible nature of the M13 template prior to metallization.

After Cu plating, the phage surfaces showed darker contrast in TEM images (Figures 6e-f and 7e-f) and a higher Cu to Pd ratio in the EDS analysis (Additional file 1: Figures S5 and S6) indicating a successful copper coating. The average diameter of the copper coated nanorods was 35 ± 8 nm for Cu-*fd* Y21M and 24 ± 4 nm for Cu-M13. The Cu-*fd* Y21M nanorods formed 2–3 μm clusters and the Cu-M13 nanorods had aggregates larger than 10 μm. Overall, based on TEM images the coating on Cu*-fd* nanorods (Figure 6e) was more uniform in comparison to Cu-M13 (Figure 7e). It is important to note that this is the first time that any metallization is reported for *fd*-Y21M.

### Synthesis of Cu-nanowires

**Figure 8:**
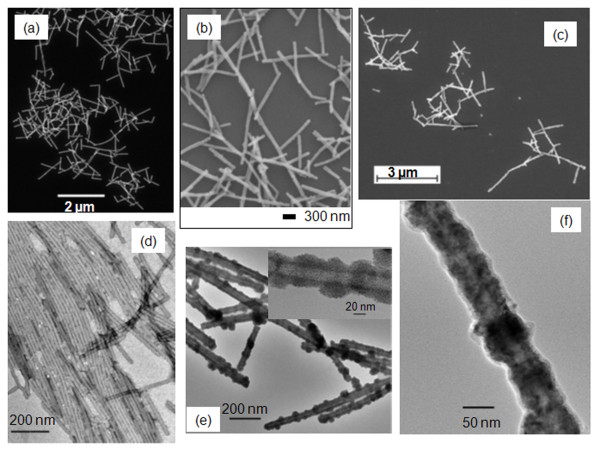
**Images of Polyanilinine (PANI)-TMV before and after metallization.** (**a**) SEM of PANI-TMV, (**b**) SEM of PANI-Pd-TMV, (**c**) SEM image of PANI-Cu-TMV, (**d**) TEM image of PANI-TMV stained with uranyl acetate (**e**) TEM image of PANI-Pd-TMV, (**f**) TEM image of PANI-Cu-TMV.

Our interest in fabricating nanostructures of high aspect ratio had motivated us to produce nanowires from polyaniline-TMV (PANI-TMV, Figure 8). Niu *et al.*[42] had shown the synthesis of PANI-TMV and studied its conductivity [53]. Our interest relies in demonstrating that our reaction conditions allow the deposition of metal on a variety of rod-like biotemplates therefore, PANI-TMV (Figure 8a and 8d) is a great candidate since the evenly distributed charges on the surface allowed Pd deposition (Figures 8b and 8e) without the need of an external reducing agent as we demonstrated in previous sections with the WT-TMV. In addition, it allows us to fabricate longer structures. One limitation of using aniline to assist the polymerization of WT-TMV is the polydispersity of the product which can be overcome by finding fractionation methods to separate the nanowires of interest. Nonetheless, the polydisperse product may find uses in certain applications.

This is the first time that PANI-TMV is coated with Pd and Cu (Figure 8) which opens the possibility of adding a variety of metals that will impart higher conductivity than the inherent conductivity that PANI-TMV has by the fact that polyaniline is a conducting polymer [53]. We had coated PANI-TMV with Cu as shown in Figures 8c and 8f. Differences in the coating can be appreciated in the TEM images (Figures 8d-f). PANI-TMV (Figure 8d) was stained with uranyl acetate for imaging purposes while PANI-Pd-TMV and PANI-Cu-TMV owe the contrast in TEM images to the metallic nature of the corresponding coating. Further experiments are in progress to: increase the thickness of the copper layer on PANI-Cu-TMV, fractionate samples for isolation of 1 μm to 2 μm size nanowires, and perform conductivity measurements.

## Conclusions

Reaction conditions described in the current work resulted to be effective in the biotemplating of a series of rod-like viruses for the fabrication of Cu-nanorods and Cu-nanowires. Cu has been successfully deposited onto the outer surfaces of TMV, *fd,* and M13 viruses, with the Cu-TMV nanorods being the straightest in the series owing to the stiff nature of the biotemplate. Furthermore, reaction conditions were developed to synthesize Pd- and Cu-nanowires of sizes longer than 1 μm by metallization of polyaniline coated WT-TMV. Excellent dispersion of individual Pd- and Cu-nanorods using complexing and capping agents has been demonstrated. By faithfully replicating the shape and dimension of the rod-like biotemplates and separating the aggregates into individual nanostructures, we had achieved an important step towards synthesizing and processing high quality Cu-nanorods and Cu-nanowires by scalable methods.

## Materials and methods

All chemicals were obtained from U.S.A. sources and used as received. Sodium tetrachloropalladate (II) (Na_2_PdCl_4_), Copper (II) sulfate (CuSO_4_), dimethylamine borane (DMAB), ethylenediaminetetraacetic acid (EDTA), Triton X-100, Sarkosyl, tetracycline, ampicillin, RNase A, and DNase I were purchased from Sigma-Aldrich (St. Louis, MO). Thiotic acid (TA), glucose, potassium phosphate, sodium chloride (NaCl), and polyethylene glycol (PEG, MW = 8000) were purchased from Fisher Scientific (Pittsburgh, PA). Kanamycin was obtained from EMD Chemicals Inc. (Philadelphia, PA), Luria Broth Base media (LB) from Invitrogen (Carlsbad, CA), and XL1-Blue *Escherichia coli* strain from Agilent Technologies Genomics (Santa Clara, CA). (1-mercaptoundec-11-yl)tri(ethylene glycol) (C11-PEG-thiol) was synthesized in our laboratory [50]. M13 and *fd* Y21M were grown and purified *in house* as described in Additional file 1. Purified wild type-tobacco mosaic virus (WT-TMV) was provided by Prof. Qian Wang’s laboratory at the University of South Carolina. All dialysis was performed against Milli-Q water at room temperature (RT) using 20 kDa molecular weight cut off dialysis cassettes from Fisher Scientific.

### Electroless deposition of Pd on WT-TMV

Pd deposition was performed by reduction of Pd^2+^ onto the TMV surface functional groups. The method was optimized for our application from a previously described protocol [27]. Briefly, WT- TMV stored in 100 mM phosphate buffer, pH 8 was dialyzed against water overnight. 2–4 μg/ml TMV was incubated with an aqueous solution of 0.4-0.8 mM Na_2_PdCl_4_ at 50°C for 30 min. This procedure produced a loosely packed brown precipitate of Pd-coated TMV (Pd-TMV).

In order to obtain individually dispersed Pd-TMV, 0.5 mM EDTA was mixed with the suspension containing Pd-TMV, and then sonicated for up to 1 hour at RT (Branson Ultrasonic Cleaner 2510). The suspension was centrifuged at 6000 rpm (3300 rcf, Eppendorf centrifuge 5415R) for 15 min at RT. After discarding the supernatant, the brown pellet containing Pd-TMV was resuspended in water. The suspension was mostly uniform in color without visible aggregation.

### Electroless deposition of Cu on Pd-TMV

Freshly prepared Pd-TMV suspension was mixed with a Cu plating solution containing 1 μg/ml TMV, 1 mM CuSO_4_, and various concentrations of dimethylamine borane (DMAB: 1 mM, 1.5 mM, 2 mM, and 3 mM) and EDTA (0.03 mM, 0.5 mM, 1 mM, and 1.5 mM) in separate experiments which were performed for optimization purposes (data not shown, optimum procedure is described herein). The pH of the plating bath was about 5. The reaction was allowed to proceed for 12 min for reaction progress study while in subsequent preparations the reaction was stopped at 6 min by diluting the reaction mix 1:1 with water. EDTA (0.5 mM in water) and TA (1 mM in ethanol : H_2_O = 8:2) were added to the suspension and centrifuged at 6000 rpm for 15 min. After discarding the supernatant, water was added to resuspend the Cu-TMV pellet. In order to further disperse the Cu-TMV, 1 μl of C11-PEG-thiol [50] was added to the suspension and incubated at RT overnight.

### Electroless deposition on *fd* and M13 phages and polyaniline-TMV

*fd* mutant Y21M was used for metallization for the first time. Mutant *fd* Y21M was selected as opposed to WT-*fd* since it has a higher persistence length in comparison to the WT-*fd*[51]. We utilized the same procedure and concentrations that produced straight and continuously covered TMV for the metallization of the phages. Namely, the Pd reactions consisted of 2 μg/ml of phage and 0.4 mM Na_2_PdCl_4_. The Cu plating baths contained 1 μg/ml of viruses coated with Pd, 1 mM CuSO_4_, 1 mM DMAB, and 0.5 mM EDTA.

PANI-Pd-TMV and PANI-Cu-TMV nanowires were synthesized by coating WT-TMV with polyaniline via aniline polymerization on self-assembled TMV rods as previously described [42]. After polymerization the sample was dialyzed against water overnight. Metallization reactions of PANI-TMV were performed using a slightly modified procedure found to be optimum for the metallization of WT-TMV. Briefly, the Pd reactions consisted of 2 μg/ml of PANI-TMV and 0.6 mM Na_2_PdCl_4_. The Cu plating baths contained 1 μg/ml of PANI-Pd-TMV, 2 mM CuSO_4_, 2.5 mM DMAB, and 1.0 mM EDTA.

### Electron microscopy analysis

Suspensions of all samples were drop casted on acid-cleaned silicon wafers and air dried in the hood. Scanning electron microscopy (SEM) of metalized TMV was performed using a Leo Supra 55 (Cal Zeiss SMT AG).

Transmission electron microscopy (TEM) was obtained from samples deposited onto a 300-mesh formvar carbon coated nickel grid (Ted Pella Inc. Redding, CA). A LIBRA-120 EFTEM equipped with an EDS detector was used for qualitative analysis of metal content (see Additional file 1 for data and discussion). Diameters of the metalized-TMV were measured and analyzed from the TEM images using Image J software (version: 1.4.3.67, copyright 1993–2006, Broken Symmetry Soft). One or two measurements were taken from each nanorod. Thirty to eighty TMV-nanorods were measured for each sample.

### X-ray photoelectron spectroscopy (XPS)

XPS was performed with a Thermo Scientific Model K-Alpha spectrometer, using the monochromatized AlKα line at 1486.6 eV. The base pressure was 1.5 x 10^-8^ Torr. The x-ray spot size was 400 μm. Argon ion sputtering for depth profiles was performed over an area of roughly 2 mm x 2 mm, using a beam energy of either 500 or 1000 eV. The data were background-subtracted and then smoothed using a Savitzky-Golay algorithm.

## Competing interests

The authors declare that they have no competing interests.

## Authors’ contributions

JCZ designed and carried metallization reactions and drafted the manuscript. CMS carried Nanosight particle size characterization, M13 production and characterization, AFM characterization of WT-TMV, and drafted the manuscript with JCZ. MC performed aniline polymerization on WT-TMV and metallization reactions in all templates and SEM characterization. MAB performed aniline polymerization on WT-TMV, TEM, and EDS characterization. MM designed, synthesized, and purified molecule for successful dispersion of metal nanorods. EB produced and purified *fd*-Y21M bacteriophage. BRR and TC conceived the study, and participated in its design and coordination. PEP performed XPS characterization and corresponding data analysis. BRS performed kinetic studies during metallization reaction. All authors read and approved the manuscript.

## Supplementary Material

Additional file 1 **Detailed experimental procedures for *fd* Y21M and M13 production and corresponding characterization methods.** EDS spectrum of Pd-nanorods and Cu-nanorods. Control experiments for determination of WT-TMV particle size distribution by Nanosight particle size determination system [51,54-58]. Click here for file
