# Supplementary material for: Biotemplating rod-like viruses for the synthesis of copper nanorods and nanowires
Source: J Nanobiotechnology. 2012 May 1;10:18. doi: 10.1186/1477-3155-10-18 (PMC3474170; doi:10.1186/1477-3155-10-18)
Supplement: Additional file 1 — Detailed experimental procedures for fd Y21M and M13 production and corresponding characterization methods. EDS spectrum of Pd-nanorods and Cu-nanorods. Control experiments for determination of WT-TMV particle size distribution by Nanosight particle size determination system [51,54-58]. [file 1477-3155-10-18-S1.doc]

**Supplementary Information**

# Biotemplating rod-like viruses for the synthesis of copper nanorods and nanowires.

Jing C. Zhou^1,5,†^, Carissa M. Soto^1*^ , Mu-San Chen^1^, Michael A. Bruckman^1, 5^, Martin H. Moore^1^, Edward Barry^2^, Banahalli R. Ratna^1^, Pehr E. Pehrsson^3^, Bradley R. Spies^4^, Tammie S. Confer^4^

^1^Naval Research Laboratory, Center for Bio/Molecular Science and Engineering, 4555 Overlook Ave. S.W. Washington, DC 20375

^2^Martin Fisher School of Physics, Brandeis University, 415 South St. Waltham, MA 02454

^3^Naval Research Laboratory Code 6100, 4555 Overlook Ave. S.W. Washington, DC 20375

^4^Naval Research Laboratory Code 5711, 4555 Overlook Ave. S.W. Washington, DC 20375

^5^NRL/NRC postdoc resident at Naval Research Laboratory, Washington, DC

*Corresponding author

^†^Current address: IBM Almaden Research Center, 650 Harry Rd. San Jose, CA 95120-6099

**Experimental**

**Preparation of *fd* Y21M phage nanoparticles**

The *fd* virus belongs to the Ff class of filamentous phages, and is therefore specific to strains of *Escherichia coli* containing the F pilus receptor [1]. XL1-Blue strain was used as the host strain for infection, and bacterial cells containing the F pilus were selected by adding tetracycline (tet) to all growth media to a final concentration of 50 μg/ml. All growth media was prepared following the recipes outlined in *Molecular Cloning* [2]. The following protocol has been adapted for the large scale synthesis of ~10^12^ particles/mL in 5 x 1 L flasks of growth media [3].

Bacterial colonies were grown on LB-tet agar plates, and isolated colonies were picked and grown overnight at 37°C under vigorous shaking (250 rpm). In the morning, 250 µl of an overnight starter culture was used to inoculate 25 ml of LB-tet media and grown to an absorbance of OD_600_ of 0.2. At this point, 2 ml was used to inoculate each of the 1 L flasks of LB-tet media. In the remaining 15 ml of bacterial suspension, 150 µl of a phage stock containing ~1x10^11^ pfu was added. When the absorbance of the phage/bacteria suspension reached an OD_600_ of 0.6, 3 ml was added to each of the 1 L flasks.

Infected cells were grown to early log phase, at which point bacterial cells were removed by two centrifugation steps (4000 x *g* for 10 min and 14,500 x *g* for 10 min). Phage particles suspended in the remaining supernatant were precipitated by adding 25 g/ ml of NaCl and PEG. Flasks containing the virus/PEG/NaCl mixtures were left at RT under constant stirring for ~30 min before centrifuging at 14,500 x *g* for 20 min to recover the precipitated particles. Phage pellets were then resuspended in dH_2_O, and further purified by two detergent treatments. The first detergent treatment subjected the phage particles to 1% Triton X-100. Phages in the presence of Triton were gently mixed for ~1 hr, precipitated using PEG/NaCl, and then resuspended after centrifugation in dH_2_O. The phages were then subjected to a second detergent treatment using 1% Sarkosyl. Phages in the presence of Sarkosyl were gently mixed for ~1 hr and then precipitated using PEG/NaCl as described above.

Purified phages were concentrated and resuspended in buffered solutions via high speed centrifugation (~250,000 x *g* for 1 hr). Concentrations of the phages were measured using absorption spectroscopy. The *fd* virus at a concentration of 1 mg/cm^3^ has an OD of 3.84 for a path length of 1 cm at 269 nm. In the case of *fd* Y21M, the OD is 3.63 to account for the lack of absorption by methionine at 269 nm [4]. All phage suspensions were stored at 4°C to limit any residual bacterial growth.

**Preparation of M13 phage**

Amplification and purification of M13 phage was performed by standard biological methods. Briefly, hundred microliters from a M13 library (XL1 Blue *E.coli* cloning strain [5]) were thawed in ice and used to inoculate 50 ml LB media to which glucose and ampicillin was previously added up to 2 % and 100 μg/ml respectively. Cells were grown by shaking at 250 rpm at 37 °C until OD_600nm_ reached 0.5. Ten ml of the culture was transferred to a 50 ml centrifuge tube and infected with 1.6 µl of helper phage (10^13^ particles/ml). Cells were incubated without shaking for 30 min at 37 °C. Infected cells were spun at 3300 x *g* for 10 min and resuspended gently with 50 ml of LB containing ampicillin (100 μg/ml) and kanamycin (25 μg/ml). Cells were grown by shaking at 250 rpm overnight at 30 °C. Cells were spun down at 3000 x *g* for 10 min. M13 phage was precipitated by mixing the supernatant with 1/5 the volume of 2.5 M NaCl/20 % PEG. The mixture was incubated in an ice/water bath for 1 hr. After 1 hr, the mixture was centrifuged for 10 min at 3000 x *g*. The supernatant was decanted and the pellet resuspend in sterile phosphate buffered saline (PBS). The resuspended phage was transferred to 1.5 ml microcentrifuge tube and centrifuged at 11000 x *g* for 5 minutes and the supernatant containing the M13 phage was transferred to clean microcentrifuge tubes.

In order to purify the M13 even further prior to metallization reactions, the phage was precipitated and treated with nucleases. Detailed procedure: 0.5 ml of the M13 phage solution was mixed with 0.1 ml of 2.5 M NaCL/20 % PEG and incubated in ice for 1 hr. Mixture was centrifuged at 3000 x *g* at °C. Supernatant was discarded and the white pellet was dissolved in 1 ml of 10 mM potassium phosphate (KP) pH 7.5. After which 10 µl of RNase A (10 mg/cm^3^) and 10 µl of DNase I (10 mg/cm^3^) were added and shaken at 250 rpm, 37°C for 30 min. 0.1 ml of 20% PEG/NaCl solution were added and incubated in ice for 15 min followed by centrifugation at 3000 x *g*. The pellet was recovered and dissolved in 1 ml of 10 mM KP pH 7.5. Samples were imaged by atomic forced microscopy (AFM) to ensure purity.

**Atomic force microscopy (AFM)**

Virus and bacteriophages were characterized using a Multimode scanning probe microscope (Veeco Instruments, Plainview, NY, USA) operated under tapping mode in air with a silicon cantilever (Nanoscience Instruments, Phoenix, AZ), 125 µm long, with an apex curvature radius <10 nm, a resonant frequency of 300 kHz, and a spring constant of 25-75 N/m. Image processing was performed using Research NanoScope II software version 7.20. All images were filtered using the flattening built-in tool from NanoScope II software. Length and height values of the nanoparticles were obtained by utilizing the built-in tool for cross section analysis. 5 µl of virus samples were placed on MgCl_2_ modified mica. Samples were dried at RT and washed for 30 sec by dipping in DI water. Excess water was wicked out using a kimwipe and dried under N_2_.

**Nanoparticle size determination system**

A NanoSight LM10-HSBF nanoparticle size determination system along with corresponding software Nanosight NTA2.1 (www.nanosight.com; Nanosight Worthington, OH, USA) was used for particles analysis in solution. NanoSight system uses laser scattering to track the Brownian motion of individual nanoparticles and calculates the hydrodynamic diameter of the nanoparticles. Since the software Nanosight NTA2.1 was designed to determine the size of spherical particles, the system cannot quantitatively report the length of rod-shaped TMV. However, it can still be used to characterize the degree of aggregation of the nanorods. The size of the TMV nanorods obtained from the measurements was used only for qualitative comparison.

Cu-TMV samples with and without C11-PEG-thiol were diluted in series with Milli Q water to reach concentrations of 10^8^ particles/ml. Results from optimum dilutions are presented. For Nanosight analysis, a 60 sec video was taken at room temperature. Video was analyzed using Nanosight NTA 2.1 software. Brightness and gain was the same for all samples and the detection threshold was automatically adjusted by the software. A blur size of 5 pixel x 5 pixel was chosen for all samples.

**Results and discussion**

**WT-TMV Characterization**

AFM characterization of WT-TMV was performed for quality control purposes. Micrographs show well dispersed particles of the expected shape and size (Figure S-1). WT-TMV was used as a control for dispersion of nanorods during nanoparticle degree of aggregation analysis.

**Figure S-1 AFM characterization of WT-TMV.** WT-TMV as received was diluted up to 0.1 mg/ml with DI water. (a), rod-like particles are observed as expected. (b) Higher magnification of image as shown in a. (c) Height profile of rods shown in b.

**WT-TMV particle size analysis with NanoSight LM10-HSBF: Control experiments**

TMV samples received from Qian Wang’s laboratory (Figure S-1) were diluted to (1-7) x 10 ^8^ particles/ml with water using serial dilution (Table S-1). For Nanosight analysis, a 60 sec video was taken at RT and analyzed using Nanosight NTA 2.1 software. Brightness and gain was the same for all samples and the detection threshold was automatically adjusted by the software. Blur size was kept 5 x 5 for all samples. It is important to note that camera level was set higher for WT-TMV samples (camera level 7) since plain WT-TMV scatters much less than Pd-TMV and Cu-TMV (camera level set to 4 for metalized-TMV). Nanosight results of Cu-TMV are described in the main text. Since the software does not account for the rod-shape of the particles quantitative size distribution cannot be obtained. These control experiments provided the size profile expected for WT-TMV which resulted in a single peak. Based on what was expected for non-aggregated WT-TMV samples (Table S1, Figure S-1), we were able to determine the level of aggregation of metalized-TMV samples. The presence of aggregates was determined by profiles that showed broader peak distribution in comparison to WT-TMV controls.

**Table S-1 Particle size distribution of WT-TMV from Nanosight analysis.**

*Average particle size: 86±10, n=6.

**Figure S-2 Representative size profile of WT-TMV from Nanosight particle sizer.** Major peak is centered at 72 nm. The lack of peaks at larger sizes indicates that the sample is monodisperse and well-dispersed.

**AFM characterization of M13 phage**

M13 particles were imaged by AFM to determine the quality of the sample after preparation. Fiber-like structures of ~ 900 nm in length are observed as expected (Figure S-3). The height of particles (2.5 nm) is smaller than the theoretical value (6.6 nm) due to the compression of the fiber on the surface. Similar behavior had been observed for TMV on MgCl_2_ modified surfaces [6].


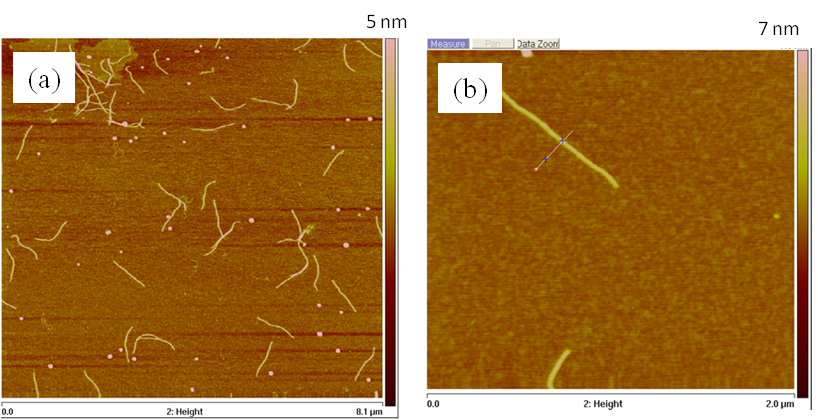


**Figure S-3 AFM of M13 phage.** M13 was diluted in water down to 10^8^ particles/ml. (a), fibers are observed as expected. (b) Higher magnification of image as shown in a, particle of 2.5 nm in height and approximate length of 900 nm.

**TEM-EDS Analysis of Pd and Cu-nanorods**

EDS analysis from TEM samples was used to qualitatively determine the presence of Pd and Cu on metalized-nanorods. As shown in Figures S4-a, S5-a, and S-6-a, the signal corresponding to Pd(Lα1) (peak centered at ~ 2.8 keV) correspond to Pd in Pd-nanorods. Background levels of Cu in Pd-nanorods and the presence of Ni are due to the formvar coated Ni TEM grid. For Cu-nanorods Cu(Kα1) signal (peak at 8 keV) had increased in all samples (Figures S4-b, S5-b, and S-6-b) in comparison to its Pd-nanorods counterparts. By comparing the peak ratio of Cu(Kα1) to Pd (Lα1) a larger content of copper in comparison to palladium is present after copper deposition. More detailed characterization of Pd and Cu species is described in the main text under XPS characterization.


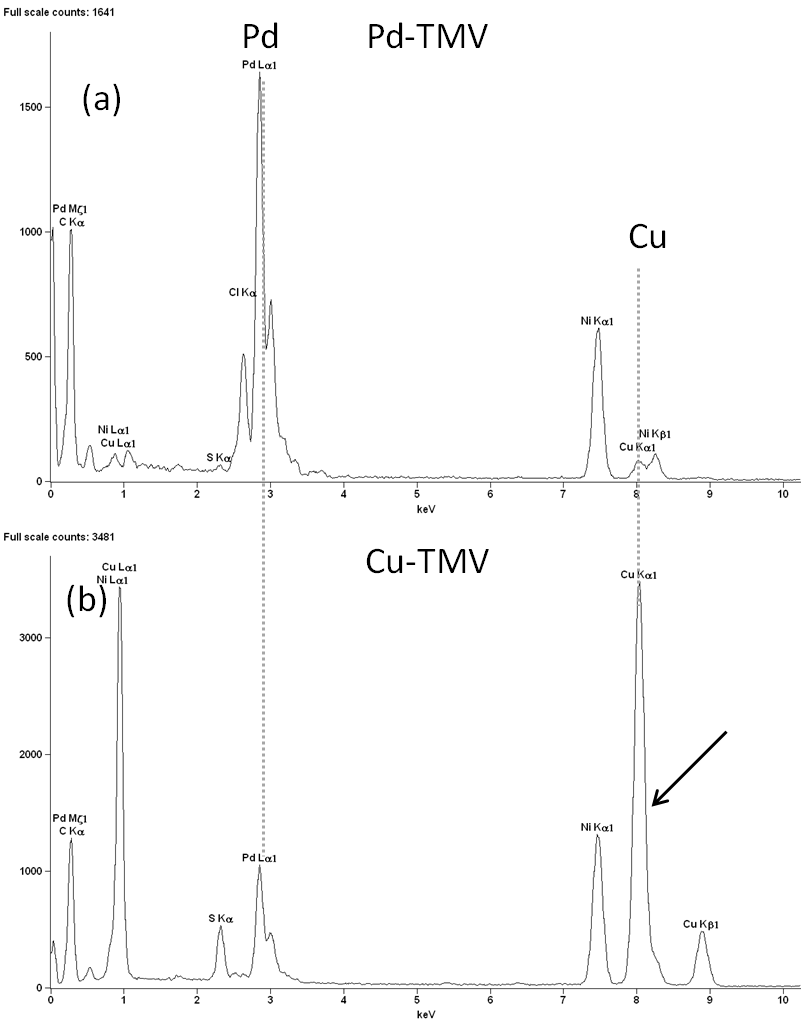


**Figure S-4 Typical EDS signals from Pd-TMV and Cu-TMV sample deposited on formvar coated Ni TEM grid.** For determining enrichment of Cu in Cu- TMV samples, Cu(Kα1) and Pd(Lα1) peaks were compared before (a) and after (b) Cu metallization. Results indicate an increase of copper content in TMV-Cu nanorods (peak denoted by arrow) in comparison to Pd- TMV sample.

**
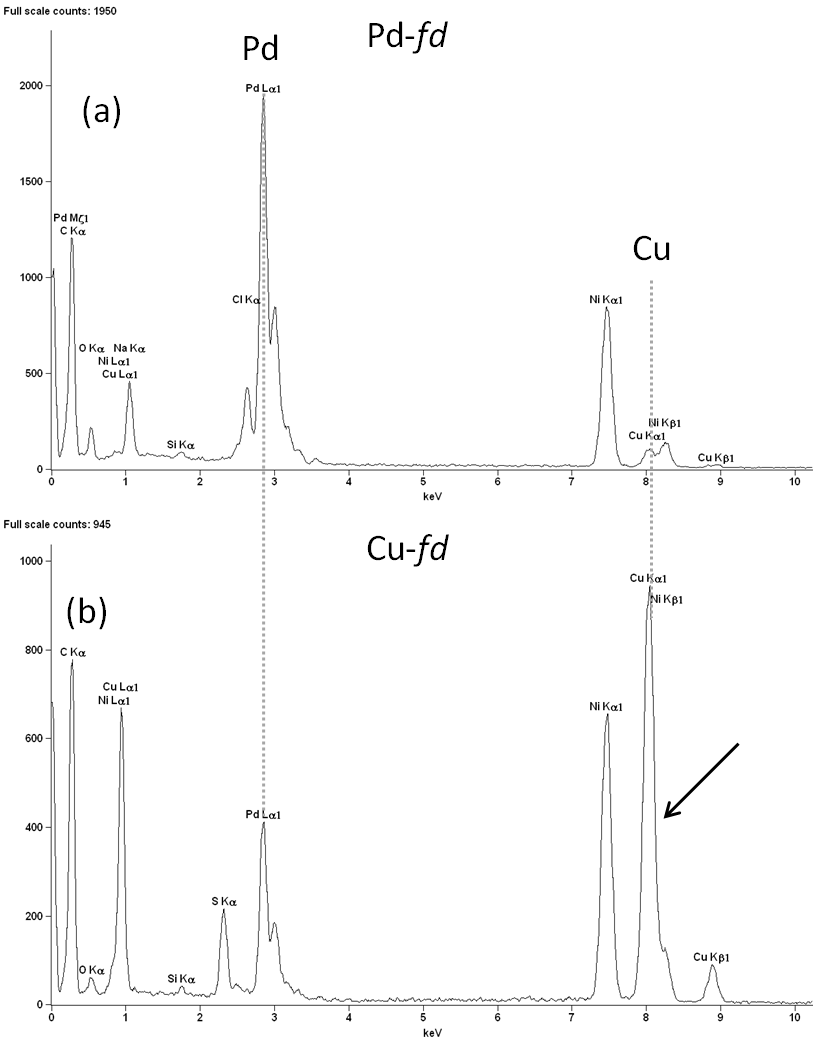
**

**Figure S-5 Representative EDS spectra from Pd-*fd* Y21M and Cu-*fd* Y21M sample deposited on formvar coated Ni TEM grid.** For determining enrichment of Cu in Cu- *fd* Y21M samples, Cu(Kα1) and Pd(Lα1) peaks were compared before (a) and after (b) Cu metallization. Results indicate an increase of copper content in Cu-*fd* Y21M nanorods (peak denoted by arrow) in comparison to Pd- *fd* Y21M sample.

**
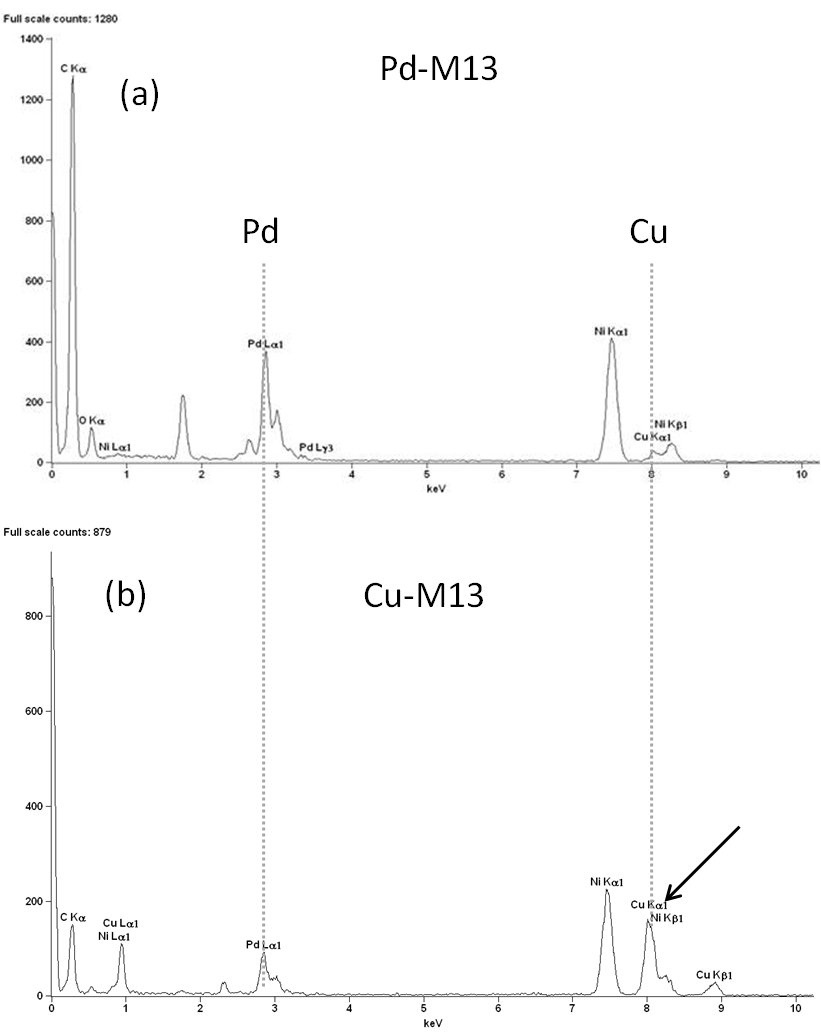
**

**Figure S-6 Typical EDS signals from a Pd-M13 and Cu-M13 samples deposited on formvar coated Ni TEM grid.** For determining enrichment of Cu in Cu-M13 samples, Cu(Kα1) and Pd(Lα1) peaks were compared before (a) and after (b) Cu metallization. Results indicate an increase of copper content in Cu-M13 nanorods (peak de noted by arrow) in comparison to Pd-M13 sample.

**References**

1. Barbas CF, Burton DR, Scott JK, Silverman GJ: *Phage display: A laboratory manual.* Cold Spring Harbor Laboratory Press; 2001.

2. Sambrook J, Fritsch EF, Maniatis T: *Molecular cloning: A laboratory Manual.* Cold Spring Harbor University Press, Cold Spring Harbor Press 1989.

3. Wickner W: **Asymmetric orientation of a phage coat protein in cytoplasmic membrane of *Escherichia-coli*.** *Proc Natl Acad Sci U S A* 1975, **72**:4749- 4753.

4. Barry E, Beller D, Dogic Z: **A model liquid crystalline system based on rodlike viruses with variable chirality and persistence length**. *Soft Matter* 2009, **5**:2563-2570.

5. Swain MD, Anderson GP, Zabetakis D, Bernstein RD, Liu JL, Sherwood LJ, Hayhurst A, Goldman ER: **Llama Derived Single Domain Antibodies for the Detection of Botulinum A Neurotoxin**. *Anal Bioanal Chem* 2010, **398**:339-348.

6. Drygin YF, Bordunova OA, Gallyamov MO, Yaminsky IV: **Atomic force microscopy examination of tobacco mosaic virus and virion RNA**. *FEBS Letters* 1998, **425**:217-221.
